# Development of Responsive Nanoparticles for Cancer Therapy

**DOI:** 10.3390/ijms241210371

**Published:** 2023-06-20

**Authors:** Jordi Puiggalí

**Affiliations:** Departament de Enginyeria Química, Universitat Politècnica de Catalunya, EEBE, Av. Eduard Maristany 10-14, E-08019 Barcelona, Spain; jordi.puiggali@upc.edu

Great efforts are focused on the development of safe nano-carriers for the treatment of cancer in order to overcome some of the typical limitations of conventional therapies. The present Special Issue collects various interesting contributions addressing the use of active nanoparticles. 

The biocompatibility, low toxicity and high therapeutic efficiency of hydroxyapatite (HAp) and carbonate apatite have justified their use as nano-carriers for drug delivery. A good example is the evaluation of goose bone ash (GBA) as a pH-responsive carrier for the delivery of doxorubicin (DOX) into breast cancer cells [1]. A prompt DOX release was observed through particle disintegration in the characteristic acidic pH of endosomal and lysosomal cancer cells. In contrast, particles remained stable under physiological conditions (i.e., pH 7.4). Cellular internalization of DOX-loaded nanoparticles was clearly higher than observed for free DOX molecules. Essentially, the surface of particles had a high affinity with transport proteins, and therefore the circulation half-life of particles in the biological environment could be increased, as well as the nanoparticle accumulation at the tumour site. 

Some new therapies are based on the fact that cancer cells are strongly anabolic and require a great amount of energy to survive and proliferate. Thus, molecules able to interfere with mitochondrial biogenesis appear especially interesting. The inhibitory effect of antibiotics against bacteria can also lead to negative effects on mitochondrial protein synthesis [2]. This feature agrees with the endosymbiosis hypothesis, which suggests that prokaryote organisms are the origin of mitochondria. The effective use of chloramphenicol (CAM) alone or in combination with other anticancer drugs has been revealed to be effective for the treatment of tumours [3]. Encapsulation of CAM into HAp nanoparticles has been considered due to their effective incorporation into tumour cells via endocytosis and their capacity to be degraded within the acidic lysosomal. Therefore, CAM can be released in the cytoplasm at a sufficient low rate to avoid the activation of typical ejection pumps. Moreover, polylactide (PLA) scaffolds incorporating CAM-loaded HAp nanoparticles have been developed, exhibiting promising properties as a drug reservoir with a high capacity to render a prolonged release depending on the polymer degradation rate [4]. Streptomycin (STR) is another antibiotic than can display a similar effect to CAM. In this way, drug-loaded amorphous and crystalline HAp nanoparticles were efficiently encapsulated in PLA electrospun scaffolds. FTIR microspectroscopy demonstrated the cytotoxic effect of STR-loaded nanoparticles when they were internalized in cancer cells [5].

Cockle-shell-derived aragonite calcium carbonate nanoparticles (ACNP) have also been evaluated as promising targeting carriers against cancer cells [6]. pH-sensitive drug release and controlled delivery of doxorubicin/thymoquinone was observed [7]. The complex system showed enhanced apoptosis, reductions in aldehyde dehydrogenase activity and in the expression of transmembrane glycoproteins, reductions in cellular migration and invasion, and finally inhibition of 3D sphere formation with respect to the use of the free drugs or the simple loaded particles. In fact, combination of anticancer drugs having different mechanisms of action is highly interesting to reduce multidrug resistance and render synergic effects. 

The efficacy of targeted drugs is impaired by their difficulty to penetrate deep into tumour tissues [8]. Thus, cell-penetrating hydrophilic small peptides (CPPs) have been explored to functionalize the surface of polymer micelles. These peptides can transport large molecules and avoid dependence on the typical endocytosis process [9]. For example, a maleinimide-tethered polyethylene glycol (PEG)-PLA block copolymer was conjugated with the transactivating transcriptional activator (TAT). Self-assembly of such compounds provided new nanoparticles able to incorporate anticancer drugs such as paclitaxel. In vitro assays demonstrated a high cytotoxic effect against human breast cancer cells because of the enhanced particle accumulation given by the activity of the small TAT peptide [10].

The pharmacological activity of nanocomposites can be improved through a deep knowledge of the cellular uptake and particle localization. The use of fluorescein isothiocyanate (FITC)-conjugated nanocomposites is interesting to identify the accumulation of particles in the cells using TEM since the success of a given treatment can be visualized. Specifically, graphene oxide–polyethylene glycol nanocarriers were loaded with protocatechuic acid as an anticancer drug, tagged with folic acid for cancer cells targeting and finally conjugated with FITC [11]. Diagnostic imaging results clearly revealed the cell uptake mechanism, changes in membrane properties and differences depending on the kind of tissue and disease condition. 

Nanoparticles can be efficiently designed to provide hyperthermia and local tumour ablation with the help of a controllable heat mediator that minimizes the adverse effects on surrounding healthy tissues [12]. Novel technologies designed to raise temperatures combine two typical procedures: the use of magnetic nanoparticles (magnetic hyperthermia), and the use of light-absorbing plasmonic nanoparticles [13]. In addition, the use of cationic liposomes as vehicles for drug delivery appears interesting due to their capability to enhance nanoparticle intracellular uptake as a consequence of their favourable electrostatic interaction with negatively charged cell membranes [14]. Thus, liposomal nanocomposites containing citric acid-coated iron oxide magnetic nanoparticles were efficient for cancer therapy induced by an alternating magnetic field (AMF) and near-infrared (NIR) lasers [15]. 

Fe_3_O_4_ has superparamagnetic properties which can be beneficial in the use of targeted therapies. Specifically, nanoparticles incorporating magnetic Fe_3_O_4_ can be directed to tumours using a magnetic field. Therefore, efforts are focused on improving the performance of such particles. A recent example is the green preparation of systems based on a montmorillonite matrix loaded with Fe_3_O_4_ nanoparticles. Carrageenan and protocatechuic acid were also incorporated acid as stabilizer and anticancer drug, respectively [16]. This nanocomposite was biologically active and showed a potential as a pH-responsive drug delivery agent.

Although research on the development and clinical applications of magnetic nanoparticles mainly concerns particles with a spherical shape, it must be considered that both magnetic behaviour and biological activity can be improved by using anisotropic nanoparticles. Magnetic drug delivery, hyperthermia and resonance imaging, among other properties, are thus dependent on the particle shape, as recently been reviewed [17]. 

The unique properties of carbon nanotubes can be considered to produce nanocarriers for both drug delivery [18] and cancer diagnosis, among other interesting applications. For example, single walled carbon nanotubes (SWCNTs) have been coupled with hyaluronic acid to improve dispersibility and coated with chlorin e6 (Ce6) to render new photosensibilized nanobiocomposite carriers for photodynamic therapy of colon cancer cells [19]. Irradiation of Ce6 at a specific wavelength induced the formation of reactive oxygen species and led to necrosis/apoptosis of cells. In general, SWCNTs can directly penetrate cells, while offering a great affinity towards photodrugs and other active agents [20]. 

Polymeric micelles have received great attention as drug delivery systems due to their biocompatibility and structural stability. Nevertheless, low drug entrapment efficiency is a great challenge. Some efforts have been focused on obtaining supramolecular micelles with hydrogen bonding motifs that are able to promote a fast self-assembly of polymeric chains to increase the affinity for selected drugs [21]. For example, interest in polypropylene glycol chains has been reported due to their difunctional uracil terminal groups (BU-PPG). The self-complementary uracil moieties are photosensitive and able to dimerize and enhance the drug (e.g., doxorubicin) loading capacity [22].

Statins are cholesterol-reducing agents, which, among other effects, seem to reduce the risk of liver cancer [23,24]. A promising therapeutic is based on the conjugation of drugs like fluvastatin (FLV) with cationic cell penetrating peptides [25]. Specifically, HIV-1 is a transactivator of transcription peptide (TAT) that can reduce the proliferation of cancer cells and serve also as an efficient carrier of FLV [26].

The highly branched architecture of dendrimers appears appropriate to develop nanocarriers due to the high number of terminal groups on their surface. Thus, they appear appropriate to bind metals with anti-cancer properties [27]. Ruthenium has a particular interest due to its cytotoxic effect and its capacity to hinder DNA replication in tumour cells [28]. Furthermore, ruthenium can be taken up by cancer cells, instead of iron, as a consequence of the rapid metabolism of such cells causing a high iron demand [29]. For example, carbosilane dendrimers functionalized with ruthenium (II) complexes have been revealed to be effective against leukaemia cells, while a low toxicity to non-cancer cells was observed [30]. These advances are significant since an intrinsic problem of leukaemia is its capacity to develop secondary resistance to chemotherapy.

The high resistance of cancer stem cells to conventional therapies impedes the eradication of cancer. Novel therapies are being developed to obtain a more effective reduction of metastasis and cancer recurrence [31]. Gold nanoparticles can be conjugated with tumour targeting agents like antibodies (Abs). These conjugates can provide cell specificity and, more interestingly, both a non-immunosuppressive character and a low tendency to cause the treatment-induced resistance of neoplastic cells [32,33]. 

Cisplatin (Cispt) is probably the most active agent against bladder cancer, since is able to form DNA adducts that lead to cell apoptosis [34]. Advantages of pegylated liposomes have been considered for loading Cispt and obtaining an extended circulation time [35]. In addition, pegylation can enhance permeation and retention effects and therefore increase the anticancer efficiency [36].

A personalized cancer treatment requires the use of ultrasensitive techniques to perform an early diagnosis. Techniques based on the use of plasmonic nanoparticles offers some advantages as a real alternative or complement to the most conventional techniques. Thus, biomarkers like specific peptides and proteins, microRNA, circulating tumour DNA and cells and exosomes can be well detected and quantified. The potential and versatility of plasmonics are revealed through the use of multiple analysis techniques like Raman scattering spectroscopy, surface plasmon spectroscopy, mass spectroscopy, dynamic light scattering spectroscopy, colorimetry and fluorescence microscopy, as has recently been reviewed [37].
